# Autonomous Robot Task Execution in Flexible Manufacturing: Integrating PDDL and Behavior Trees in ARIAC 2023

**DOI:** 10.3390/biomimetics9100612

**Published:** 2024-10-10

**Authors:** Ruikai Liu, Guangxi Wan, Maowei Jiang, Haojie Chen, Peng Zeng

**Affiliations:** 1 State Key Laboratory of Robotics, Shenyang Institute of Automation, Chinese Academy of Sciences, Shenyang 110016, China; liuruikai@sia.cn (R.L.);; 2 Key Laboratory of Networked Control Systems, Chinese Academy of Sciences, Shenyang 110016, China; 3 Institutes for Robotics and Intelligent Manufacturing, Chinese Academy of Sciences, Shenyang 110169, China; 4 University of Chinese Academy of Sciences, Beijing 100049, China; 5 Institute of Future Human Habitats, Tsinghua Shenzhen International Graduate School, University Town of Shenzhen, Nanshan District, Shenzhen 518055, China

**Keywords:** agent, robotics, agility, PDDL, behavior tree

## Abstract

The Agile Robotics for Industrial Automation Competition (ARIAC) was established to advance flexible manufacturing, aiming to increase the agility of robotic assembly systems in unstructured and dynamic industrial environments. ARIAC 2023 introduced eight agility challenges involving faulty parts, flipped parts, faulty grippers, robot malfunctions, sensor blackouts, high-priority orders, insufficient parts, and human safety. Given the unpredictability of these scenarios, it is impractical to develop a specific strategy for each possible situation. To address these issues, this paper presents a hierarchical framework for autonomous robotic task generation and execution in dynamic scenarios. The framework is divided into a task level and an execution level. Initially, an immediate task management strategy is adopted at the task level, which reasonably decomposes dynamic tasks and allocates short-term tasks to the floor robot and ceiling robot. Later, at the execution level, each robot is designed with an agent architecture that combines PDDL planning with the quick response of behavior trees. Finally, the effectiveness and practicality of the proposed framework were thoroughly validated in ARIAC 2023.

## 1. Introduction

Over the past few decades, robots have been extensively employed in manufacturing systems, primarily for performing repetitive and simple tasks. The globalized market requires manufacturing systems to have enhanced flexibility and agility, enabling them to maintain competitiveness and adaptability in ever-changing environments [1]. However, owing to their task specificity, the efficiency of traditional robot programming methods is limited under constantly changing products and events. For example, when a robot performs assembly tasks in a warehouse, it must adjust its actions on the basis of the type of object being handled (such as pumps or batteries); this typically requires the input of robot experts, which is both time-consuming and lacks flexibility [2]. To meet evolving demands, new approaches to robot agility and adaptability are needed. An initiative to facilitate the development of new approaches is the Agile Robot Industrial Automation Competition (ARIAC), which was designed to push the boundaries of robotic systems by emphasizing not only task efficiency but also the system’s ability to adapt in dynamic environments [3]. In addition to participants being required to complete specified assembly tasks in as little time and expense as possible, ARIAC 2023 introduces eight agility challenges involving faulty parts, flipped parts, faulty grippers, robot malfunctions, sensor blackouts, high-priority orders, insufficient parts, and human safety. These challenges occur randomly to simulate as closely as possible the problems that can occur in real production. These challenges occur randomly, further simulating the unpredictability of real-world manufacturing scenarios and highlighting the need for flexible, adaptive robotic solutions.

Traditional robot programming methods, while effective for repetitive tasks, often fall short in highly dynamic environments [4]. Automated planning [5,6] and machine learning [7] have emerged as the two mainstream methods for robotic task planning, offering more flexibility than static programming. However, due to the ARIAC robot system design principle of “preparing mostly for errors instead of normal operation” [8], these methods cannot provide timely solutions in dynamic and frequently faulty environments. Therefore, most action planning work by past ARIAC participants has resulted in static plans with designed rules to adapt to dynamic environments. Team RuBot used finite state machines (FSMs) to design static plans for each robot and added simple recovery modules to handle robot faults [9]. Team Reaper constructed multiple agents on the basis of task functions, reducing system coupling but still requiring static plans and rules for each agent [2]. However, these methods inevitably reduce the autonomy and robustness of robotic systems, decreasing their adaptability to dynamic tasks and environments.

To address these limitations, hybrid architectures that combine automated planning, machine learning, and control frameworks have been proposed to improve system adaptability. Recent studies in areas such as human–robot interaction, autonomous systems, and brain–computer interfaces have demonstrated the effectiveness of hybrid models in dynamic environments, offering insights into how these techniques could enhance robotic systems [10,11,12]. In recent years, behavior trees have emerged as a key component of such hybrid architectures, recognized for their superior reactivity in robotic task execution [13]. Behavior trees provide a modular framework for organizing and executing tasks, wherein nodes (including conditions, actions, etc.) define how a robot makes decisions and responds to varying scenarios. The principal advantage of behavior trees lies in their ability to react swiftly, adapting quickly to changing environments [14]. However, a single behavior tree structure is often unable to meet the diverse requirements of complex and dynamic manufacturing environments. In this context, the role of the Planning Domain Definition Language (PDDL) [15] is particularly crucial. PDDL is a language designed to describe and automatically generate task plans based on the current task and environment. By combining PDDL with behavior trees, systems can flexibly generate and adapt behavior trees in response to complex, dynamic environments. Similar studies have attempted to combine behavior trees with various planning algorithms, using knowledge base data to generate long-term plans. However, generating long-term plans in highly dynamic and complex environments is impractical. For example, although systems such as PlanSys2 [16] and BeBOP [17] have made progress in planning and execution, they still face challenges in adapting to environmental changes and real-time responses. The most relevant to our work is [18]; however, our work emphasizes rapid adaptation to dynamic challenges rather than ontology reasoning.

Therefore, a hierarchical robotics task framework for dynamic and complex environments is proposed. The framework is divided into task level and execution level. At the task level, an immediate task management strategy is adopted. This strategy is capable of providing agents with reasonable short-term tasks in the context of complex and uncertain long-term dynamic tasks, allowing them to plan quickly. As detailed in Section 3.3.1, the planning instance demonstrates the efficiency of this approach, with planning solutions being generated in approximately 0.01 s. At the execution level, each robot is designed with an agent architecture that combines PDDL and behavior trees. This architecture leverages PDDL’s symbolic planning capabilities to generate task plans while utilizing the modularity and flexibility of behavior trees to execute these plans. While the behavior tree handles the sequence of task actions, a fault monitoring mechanism is introduced. When the agent detects a fault during task execution, the system can automatically amend the behavior tree on the basis of the fault information, enabling timely responses to ARIAC’s random agility challenges.

This work won first place in ARIAC 2023, which validated the effectiveness and practicality of this system framework in that environment. The code is available at https://github.com/LKmubihei/ARIAC_paper_code, (accessed on 8 August 2024). The main highlights are as follows:

1. A hierarchical robotics task framework for flexible manufacturing that decomposes long-term tasks and assigns them to multiple robots for execution.
2. A robust agent architecture is designed to automatically generate and update the behavior tree on the basis of real-time information, thus ensuring the reliability of task execution in dynamic scenarios.
3. The proposed robotics task framework is validated on a simulation manufacturing platform, ARIAC 2023.

The remainder of this paper is organized as follows: Section 2 presents the challenges introduced by ARIAC 2023 and related work in this domain. Section 3 analyzes the dynamic task management strategy and the agent construction methodology in detail. Section 4 presents the system implementation and experimental validation. Finally, Section 5 presents the competition experience and concludes this paper.

## 2. ARIAC 2023

### 2.1. ARIAC Introduction

The ARIAC, initiated in 2017, was created to increase the agility of industrial robotic systems, aiming to improve their assembly capabilities in unstructured and dynamic industrial settings. The engineers behind ARIAC convert challenges encountered in real-world production environments into tasks within the Gazebo simulation platform. The participants are required to develop agile robotic control systems based on the robot operating system (ROS), which is capable of handling the dynamic tasks set forth by the ARIAC [3]. In this competition, robot agility can be specifically represented by the following traits. (1) Failure recovery: robots can detect and fix issues during manufacturing. (2) Automated planning: robots require less setup time when handling new products. (3) Flexible environments: robots adapt to parts in different positions. (4) Plug-and-play robots: different robots work seamlessly without requiring major reprogramming.

**Figure 1:**
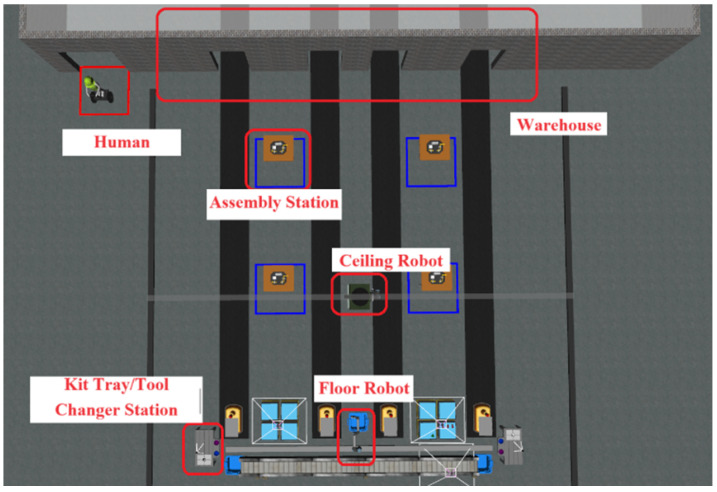
The ARIAC 2023 environment.

In ARIAC 2023, participants were required to complete dynamically issued orders, which encompass three types of tasks: kitting, assembly, and combined tasks [19]. As illustrated in Figure 1, these tasks require participants to control floor and ceiling robots to precisely select specific parts from conveyors or bins, including different colored pumps, batteries, regulators, and sensors, and move these parts to designated locations.

For the kitting task, the robot is required to place the scattered parts of an order into a kit. Specifically, the robot must pick the selected parts, place them in the designated areas of the kit tray, and use an automated guided vehicle (AGV) to transport the tray to the warehouse. The completed kit trays were scored based on accuracy and completeness. The assembly task requires the robot to assemble parts onto products at the assembly station workbench following the manufacturing process in sequential or arbitrary order. At the beginning of the task, parts are typically already placed on the AGV. Participants must move the AGV to the correct assembly site and perform the assembly there. Upon completion, the assembled products are evaluated and scored based on quality and accuracy. The combined task is an integrated task requiring participants to complete the kitting task and assembly task in sequence. Since the assembly part is scored, participants can flexibly adjust their strategies to smoothly complete the combined task.

During task execution, the competition system introduces agility challenges to test the agility of the robotic systems, with the specific challenges displayed in Table 1. The competition results account for cost factors (cost of system sensors), efficiency factors (time taken to complete tasks), and completion scores (accuracy of task execution) when assessing the performance of the participants.

### 2.2. New Trends in ARIAC 2023

Migration from ROS1 to ROS2. In 2023, ARIAC updated its implementation architecture from ROS1 to ROS2 [20] to align the competition with technological advancements. Although ROS1 and ROS2 do not significantly differ in terms of robotic control architecture implementation, ROS2 offers considerable advantages in real-time performance, security, and decentralization, which positions it as the new standard in robotics technology. Consequently, choosing ROS2 as the competition environment makes ARIAC take technological progress into account and presents a new challenge for participants: designing control systems that are more compatible with the ROS2 architecture to fully leverage its advanced features.

Task Complications. ARIAC 2023 introduced combined tasks that require the execution of assembly and kitting tasks. This increases the system complexity, requiring competitors to effectively manage two different types of tasks and complete them within a limited amount of time. This demands that the system has strong perception and planning capabilities to manage and utilize available resources effectively, completing tasks in an optimal manner.

Randomization of Agility Challenges. In ARIAC 2023, the conditions for triggering agility challenges changed. In addition to the original triggers from various events (such as placing parts and submitting orders), time-based triggers were added, making the occurrence of challenges unpredictable and random. This temporary uncertainty increases the complexity and unpredictability of the competition. These changes make the competitive environment more similar to real industrial production settings, increasing the demands on the adaptability, response speed, and decision-making capabilities of the participating systems.

### 2.3. Related Work

Team RuBot [9] shared their experience participating in ARIAC 2019 and ARIAC 2020. The robotic control system was divided into a high-level task planning layer and a low-level motion planning layer. The high-level layer uses a finite state machine (FSM) for robot task allocation, constructing precise planning paths through meticulous design at the lower level. To address agility challenges, Team RuBot designed simple recovery modules in the state machine to respond to emergencies. These modules enabled the robot to shut off its vacuum gripper and return to its original position if it became stuck or frozen for a sufficient length of time, addressing more than half of the system failures. This design highlights the importance of incorporating recovery modules into robotic systems, although indiscriminate recovery approaches can severely impact system execution efficiency. Team Virsli [8], the winners of ARIAC 2020, structured their robotic control system into four layers: strategic planning, trajectory planning, low-level robot control, and perception. When system failures occurred during agility challenges, responses were managed through the top-level strategic planner. Specifically, the strategic planning layer utilized a stateless microservice architecture, decomposing the program into a series of small, independent services. These services, which were not reliant on previous system states, allowed the system to flexibly formulate new action plans upon detecting failures caused by agility challenges. In ARIAC 2021, Team Reaper employed an agent-based architecture [2], dividing the agents into four types according to the functional requirements of the ARIAC: the order agent, task agent, robot agent, and sensor agent. They organized the multiagent system structure using Unified Modeling Language (UML) and controlled the dynamic behavior of agents through state machine transitions. Under an instant task assignment strategy, upon detecting agility challenges, the system could swiftly switch states or generate new plans through the division of labor and collaboration among agents.

Although the aforementioned methods address the adaptability of robotic systems in dynamic environments to varying degrees, they all share several common shortcomings: Response delay: Predefined static state machines and high-level strategic planning can hardly cover all unexpected situations, resulting in a slow response or even execution failure in dynamic environments. Difficulty in expansion: Recovery modules and stateless microservice architectures lack flexible adaptive capabilities when responding to complex and changing environments, making it more difficult to expand the system and handle unforeseen failures.

In this paper, a hierarchical robotics task framework is proposed to autonomously complete ARIAC order tasks and promptly respond to dynamic agility challenges.

## 3. Hierarchical Robotics Task Framework

### 3.1. Overview

Building on the experience obtained from winning ARIAC 2021 [2], we explored in depth the advantages of agent-based architectures in addressing agility challenges. An agent-based architecture offers a modular system design, enabling the decentralized design of autonomous and cooperative systems. The excellent flexibility and robustness of this architecture make it ideal for handling dynamic and unpredictable environments. For the joint tasks performed by the floor robot and ceiling robot in ARIAC 2023, we considered treating each robotic entity as an agent and controlling it with an agent design that integrates behavior trees and PDDL. This design approach allows each robot to handle ARIAC’s dynamic tasks autonomously and flexibly while ensuring reactivity.

**Figure 2:**
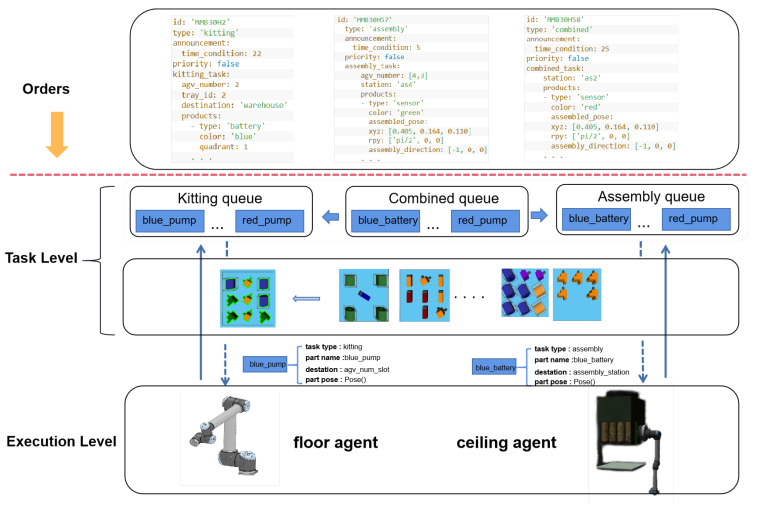
Hierarchical robotic task framework: The CCS dynamically announces orders. The system controls the floor robot and ceiling robot to fulfill these dynamic orders. The floor robot is a UR10e arm mounted on a linear rail, and the ceiling robot is mounted to a gantry on the ceiling and can move along the x- and y-axes and rotate.

Experience from ARIAC 2021 shows that using instant task assignment strategies is appropriate when handling highly uncertain, rapidly changing environments. We constructed a dynamic task manager to allocate and coordinate tasks for the two agents in real-time, thus achieving a hierarchical robotic task framework that can effectively address the dynamic task and agility challenges in ARIAC 2023. As shown in Figure 2, the framework is divided into a task level and an execution level, which are designed to handle orders from the competitor control system (CCS). At the task level, the task manager first receives the order and breaks it down into multiple parts. It then distributes these parts to three specialized task queues: kitting, assembly, and combined. The execution level is composed of a floor agent and a ceiling agent. The floor agent and ceiling agent extract individual part tasks from the relevant task queues, obtain the specific part positions through the sensor system, and execute these tasks in a closed-loop manner.

### 3.2. Task Level—Dynamic Task Management Strategy

The task level primarily consists of a task manager, which is the central component responsible for managing the workflow and task execution within the system. It is intricately connected with the CCS to ensure seamless order processing. One key function of the task layer is to subscribe to real-time orders from the CCS. This subscription mechanism allows the system to receive and process incoming orders promptly. Upon receiving these orders, the task manager of the task layer decomposes the orders into individual part tasks, which serve the smallest unit. Then, depending on the type of order, these individual part tasks are added to different task queues. Additionally, the task layer actively cooperates with the CCS to submit completed orders. Once a group of part tasks in an order is successfully executed, the task layer notifies the CCS that the order task has been completed. This interaction is crucial for maintaining accurate records of completed tasks and ensuring the smooth operation of the entire order fulfillment process.

Owing to the significant uncertainty caused by agility challenges in ARIAC, it is difficult to achieve task optimization and allocation for robots via global planning algorithms. The immediate strategy proposed by [2] is suitable for solving ARIAC tasks. On this basis, we have also devised a rule-based task allocation method.

First, in the task system, the task manager breaks down the received orders into individual tasks. This fine-grained task decomposition is key to achieving real-time task allocation, making the system more flexible and allowing it to respond more quickly to changes in sequential tasks. The decomposed tasks are distributed into three queues on the basis of their type: kitting, assembly, and combined. Each part is treated as the smallest unit of a task and is sorted according to the priority of the part tasks. Task interchangeability between different queues is also allowed. For example, a combined part task can be decomposed into kitting and assembly tasks, where the target part is first picked and placed on an AGV, which then transports it to the assembly station for assembly. Alternatively, the task can be directly converted into an assembly task, where the ceiling agent picks the target part directly from a bin or conveyor belt and transports it to the assembly station for assembly. This task decomposition and conversion mechanism enables the system to respond flexibly to various task demands, thereby improving overall efficiency.

The floor agent can complete kitting tasks only, whereas the ceiling agent can complete kitting, assembly, and combined tasks. To achieve the goal of minimizing the completion time of ARIAC tasks, the task acquisition method is based on the following rules:

1. When the floor agent is idle and functioning normally, it retrieves parts from the kitting queue. When the ceiling agent is idle and functioning normally, it retrieves parts from the queues in the order of assembly, combined, and kitting.
2. Combined tasks are decomposed into kitting tasks and assembly tasks when both the floor and ceiling agents are idle. In other cases, combined tasks are directly converted into assembly tasks.
3. Tasks involving parts on the conveyor belt are prioritized for completion.

### 3.3. Execution Level—Agent Architecture

**Figure 3:**
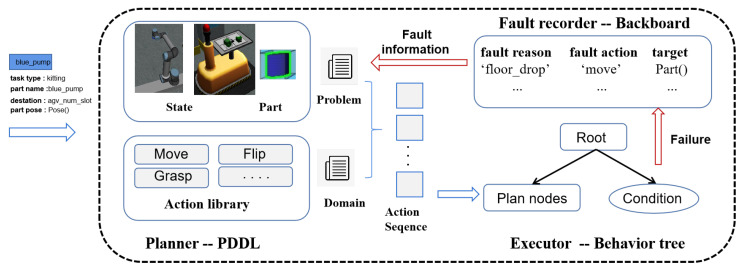
Diagram of the proposed agent architecture. The agent is composed of a planner, an actuator, and a fault recorder.

The execution level consists of two agents, the floor agent and the ceiling agent, which receive individual part tasks from the task manager. As depicted in Figure 3, the agent architecture is divided into three parts: the planner, executor, and fault recorder. The planner uses PDDL to construct behavior trees for specific tasks, enabling the agent to handle various tasks. The executor implements these behavior trees, quickly identifying and responding to agility challenges. The fault recorder logs fault information during execution failure, which is used by the planner for subsequent planning sessions. Successful task execution triggers a success signal, allowing the agent to proceed to the next task, whereas failure sends a failure signal to the task manager, records fault information on the blackboard, and returns the task to the queue for reassignment. This closed-loop execution is essential for achieving system agility.

In this architecture, the core of each agent is its action library, which serves as a shared knowledge resource for both PDDL and behavior trees. This structured design simplifies the construction of intelligent agents, as designers only need to specify the action library to quickly build an agent. There is no need for designers to write complex code for all possible scenarios; instead, they only need to create a domain file based on the action library in advance. PDDL automatically extracts data from the current environment to generate problem files and converts the generated plan into a series of actions in a behavior tree to effectively complete the assigned tasks.

The control architecture that integrates PDDL and behavior trees, as discussed in the literature [18], was designed for single-robot tasks and relies on a specific reasoning framework. When a fault occurs, this architecture updates the motion planning and TAMP configuration files to generate a new behavior tree, which increases complexity and computation time. In contrast, our proposed framework decouples planning and execution. The planning module generates PDDL problem files from the current environment and focuses on symbolic planning, while the execution module handles task execution. If an error is detected during execution, monitoring nodes interrupt the execution, return a failure signal, and reassign the task to another robot, ensuring task continuity and reducing system downtime. This architecture is especially effective in environments like ARIAC, which are designed to “prepare for errors rather than normal operations”, offering robustness and high task completion rates.

Because the architecture uses relatively independent modules, in the event of execution failure, the behavior tree will only return a failure signal, and specific fault information might be overlooked during task replanning, making it unusable. This can be critical for some failures. For example, during replanning, if a part has already been grasped and placed on the gripper, a failure might occur. Although the replanning process accounts for the presence of an object on the gripper, the object’s parameters are not returned. Consequently, the system identifies a new part on the basis of the target and sensor system, forcing the robot to find a new part among the available parts and use its parameters for the task. To address this issue, we designed a fault recorder to log the corresponding fault information. When the planner next performs planning, it attempts to extract parameters from the fault information and assigns them to the planner on the basis of the fault conditions.

#### 3.3.1. Agent Planner—PDDL

PDDL is crucial for ensuring the adaptability of agents by acting as the planner within the agent structure. PDDL is responsible for perceiving and understanding the environment and for making final decisions. In this process, the domain description defines the objects to be specified in the domain, predicates on the basis of these objects, and defines actions that produce certain effects under specific preconditions. The problem description discusses the specific instances of these objects, which predicates are true, and the goals that must be achieved [21].

PDDL has been actively developed and used in AI planning for more than two decades; multiple versions have been developed, including PDDL2.1 [22], PDDL3.1 [23], and PDDL+ [24]. Although the planning problems in ARIAC involve numerous variables, at the individual agent level, only the action sequencing problem must be examined. The expressive power of the STRIPS [25] is sufficient for this purpose, allowing for a more flexible choice of the PDDL version. Utilizing PDDL as the planner of an agent allows the problem domain, states, and goals to be clearly defined, enabling the system to autonomously understand tasks and devise appropriate action plans. Let us consider a simple example of the utilization of PDDL in ARIAC 2023.

The domain in Listing 1, named ‘ariac_domain’, provides the basic framework and rule definitions for the planning problem. It describes the types of objects available in the system, such as containers, parts, and robots, and their relationships, represented by a series of predicates, such as ‘attach’, ‘is_enabled’, ‘is_reachable’, ‘on ’ and ‘flip_on’, to describe the system state and connections. Additionally, this file defines a series of actions, such as ‘flip’, ‘grasp’, ‘move’ and ‘place’, each with its own preconditions and effects, dictating the timing and manner of execution of these actions. Defining rules and constraints for the problem enables the planner to effectively find solutions to the problem.

**Listing 1.** PDDL domain description.
(define (domain ariac_domain)

 (:requirements :strips :typing)

 (:types container part robot)

 (:predicates (attach?part - part?robot - robot)

 (is_enabled?robot - robot)

 (is_reachable?robot - robot?container - container)

 (on?part - part?container - container)

 (flip_on?part - part?container - container))

 (:action flip

 :parameters (?robot - robot?part - part?container - container)

 :precondition (and (is_enabled?robot)

 (is_reachable?robot?container)

 (flip_on?part?container))

 :effect (on?part?container)

 )

 (:action grasp

 :parameters (?robot - robot?part - part?container - container)

 :precondition (and (on?part?container)

 (is_enabled?robot)

 (is_reachable?robot?container)

 (not (attach?part?robot)))

 :effect (attach?part?robot)

 )

 (:action move

 :parameters (?robot - robot?source_container - container?destination_container - container)

 :precondition (and (is_enabled?robot)

 (is_reachable?robot?source_container))

 :effect (and (not (is_reachable?robot?source_container))

 (is_reachable?robot?destination_container))

 )

 (:action place

 :parameters (?robot - robot?part - part?container - container)

 :precondition (and (is_enabled?robot)

 (is_reachable?robot?container)

 (attach?part?robot))

 :effect (and (not (attach?part?robot)) (on?part?container))

 )

)


The problem in Listing 2, named ‘ariac_floor_problem’, specifies a planning problem within the context of the “ariac_domain” planning domain. It defines various objects, including containers, parts, and a robot (floor_robot), along with their initial configurations. The initial state includes information about the robot’s activation, its position, and the locations of parts and containers. This problem must be solved to achieve a specific task: placing a particular part (battery_red) onto a specific container (agv1). This problem file outlines a concrete planning challenge where a planner must find a sequence of actions to accomplish the task, transitioning from the initial state to the desired goal state.

**Listing 2.** PDDL problem description.
(define (problem ariac_floor_problem)

 (:domain ariac_domain)

 (:requirements :strips :typing)

 (:objects agv1 - container agv2 - container agv3 - container

 agv4 - container battery_blue - part battery_green - part

 battery_orange - part battery_purple - part battery_red - part

 bin1 - container bin2 - container bin5 - container

 bin6 - container curr_position - container floor_robot - robot

 pump_blue - part pump_green - part pump_orange - part

 pump_purple - part pump_red - part regulator_blue - part

 regulator_green - part regulator_orange - part regulator_purple - part

 regulator_red - part sensor_blue - part sensor_green - part

 sensor_orange - part sensor_purple - part sensor_red - part)

 (:init (is_enabled floor_robot)

 (is_reachable floor_robot curr_position)

 (on battery_red bin1)

 (on pump_red bin1)

 (on regulator_green bin2)

 (on sensor_green bin2))

 (:goal (on battery_red agv1))

)


Listing 3 provides the solution to the PDDL planning problem. In this solution, the robot (floor_robot) initially moves from its current position (curr_position) to container bin1. Afterward, a part named battery_red is grasped from bin1, this part is moved from bin1 to agv1, and the part is finally placed into agv1. The sequence of these actions forms the plan that successfully transitions the problem from its initial state to the desired goal state.

**Listing 3.** PDDL solution.
Time: 0.013570785522460938s

Plan:

move floor robot curr position bin1

grasp floor robot battery red bin1

move floor robot bin1 agv1

place floor robot battery red agv1


PDDL solvers exhibit a slow generation speed when handling complex, multistep global plans, which has long been a point of criticism [8]. However, in our research framework, the application and objectives of PDDL have significantly improved. PDDL has demonstrated unique advantages, particularly when addressing tasks in the dynamic environment of ARIAC. The distributed framework employed in this study allows agents to focus on tasks involving single parts rather than complex overall plans. This approach effectively reduces the risk of planning failures and enhances the adaptability of agents in dynamic environments. In traditional applications such as Plansys2 [16], PDDL is used to generate global plans involving complex actions, often encompassing numerous steps and times. However, in our framework, PDDL is utilized to generate action sequences for individual part tasks. As shown in the results of Listing 3, because fewer planning steps are involved, the speed of plan generation is significantly higher; the framework thereby overcomes the speed limitations of PDDL. This ability to quickly generate plans for individual tasks greatly enhances the agents’ response speed and real-time decision-making capabilities.

#### 3.3.2. Agent Executor—Behavior Tree

In agent architecture, behavior trees function akin to ‘hands’ and are responsible for executing specific actions and tasks. The advantage of this architecture lies in its excellent reactivity, which enables agents to respond quickly to agility challenges and dynamic tasks. Additionally, the modular structure of behavior trees allows for effective mapping within the action library, facilitating the construction and maintenance of agents.

Behavior trees were originally applied in computer games [26]. Michele et al. [27] considered the application of behavior trees in robot control and proposed the classic behavior tree framework. Owing to its adaptability and responsiveness, this framework has become popular in robot control [28]. A behavior tree consists of a root node, control flow nodes (sequence, fallback, or parallel), and execution nodes (action or condition) (see Table 2). The tick signal starts at the root node and flows from left to right through the tree. Each leaf node returns one of three states: success, failure, or running.

Notably, different behavior tree libraries or tools might introduce variations in the expression and functionalities of behavior trees. In the open-source tool py-trees (https://py-trees.readthedocs.io/en/devel/, accessed on 8 August 2024 ), the fallback node is replaced by a selector node, and the sequence node has memory and non-memory variants. Parallel nodes are also divided into selective parallel and sequential parallel types. These varied functionalities enhance the flexibility of py-trees in organizing the action structure.

Reactivity is a crucial feature of modern Behavior Tree frameworks [29], allowing agents to adapt flexibly to changes in the external environment. In particular, the recurrent execution mechanism of the root node (recurrent ticks) is crucial for enhancing the agent’s responsiveness. The root node periodically sends a looping ‘tick’ signal throughout the behavior tree, continuously triggering a reevaluation. This allows the agent to regularly check and adjust its behavior to adapt to environmental changes. The frequency of the recurrent execution of a root node can be adjusted according to specific needs, meeting various response time requirements.

Behavior trees exhibit significant modularity in the design of agent behaviors [30]. Behavior trees use a uniform task interface, standardized return states (success, failure, running), and techniques like hierarchical structures, composite nodes, and blackboards. These features improve the maintainability and scalability of agent behaviors. For example, the fault recorder employs blackboard technology. Upon receiving a task failure signal, it parametrically stores the current information, including the fault reason, the failed action, and the current goal, on the global blackboard. When the planner reinitiates the planning process, it attempts to extract parameters from this fault information and allocate them to the planner accordingly.

In the dynamic competition environment of ARIAC, when detecting agility challenges, behavior trees enable agents to handle various emergencies effectively through their flexibly configured conditional nodes. Designers can customize the settings of these conditional nodes according to specific needs and environmental changes. Since behavior trees are executed cyclically at a set frequency, the behavior of an agent can be quickly interrupted or altered when the preset conditions of a certain conditional node are no longer met. This mechanism allows agents to keenly monitor and respond to changes in the environment and the robot’s own abnormal states, thereby maintaining efficient operations in a dynamic environment.

## 4. System Implementation and Experimental Validation

### 4.1. System Implementation

In ARIAC 2023, the system employed a multinode distributed communication framework based on ROS2. In this framework, each agent is designed as an independent node, thereby yielding a high degree of modularity and parallel processing capabilities. These agent nodes are coordinated through a centralized master node, ensuring unified management and efficient operation of the entire multiagent system.

Upon receiving dynamic task orders from the CCS, the task manager assigns individual tasks to each agent. These tasks are first represented using the PDDL, where the problem is formulated by defining objects, predicates, initial conditions, and goal states relevant to the task at hand, as outlined in Section 3.3.1. The PDDL-related libraries (pddl-parser, https://github.com/pucrs-automated-planning/pddl-parser, (accessed on 8 August 2024), AI-Planning/pddl, https://github.com/AI-Planning/pddl, (accessed on 8 August 2024)) in Python were employed to generate a detailed action plan based on this task description.

Once the task has been planned via PDDL, the generated action sequence is transformed into an executable behavior tree using the PyTrees library. The behavior tree organizes the task into a modular and hierarchical structure, where each node corresponds to an action or condition. This behavior tree framework enables closed-loop task execution, allowing agents to continuously monitor and adapt to real-time changes in the environment. As the agents execute their behavior trees, they react dynamically to system states and adjust their actions accordingly. In the event of failure, such as a robot malfunction, the failure state is detected, and the task and failure reason is returned to the task manager for reassignment.

**Figure 4:**
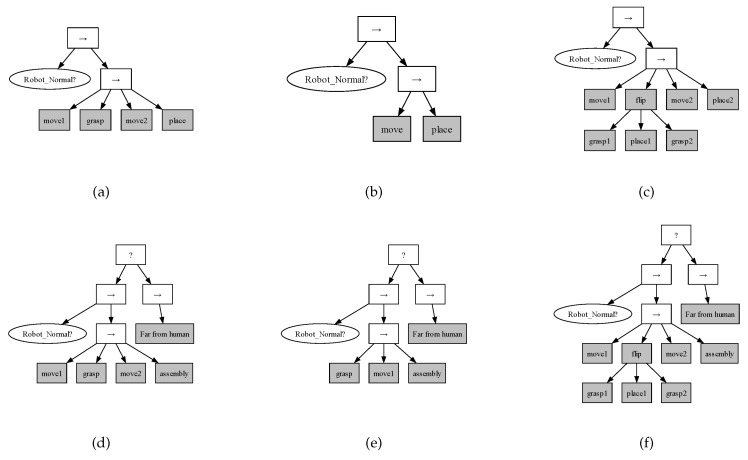
Behavior trees for various tasks and scenarios. (**a**) Floor robot behavior tree for normal kitting tasks. (**b**) Floor robot behavior tree after robot malfunction recovery. (**c**) Floor robot behavior tree for the flipped part. (**d**) Ceiling robot behavior tree for normal assembly tasks. (**e**) Ceiling robot behavior tree after gripper malfunction recovery. (**f**) Ceiling robot behavior tree for the flipped part.

Figure 4 illustrates examples of the behavior trees for both floor and ceiling robots under various task scenarios in the ARIAC environment. These examples highlight the flexibility and adaptability of the proposed task framework, which enables robots to respond efficiently to dynamic changes and task failures.

Figure 4a shows the behavior tree for a floor robot performing a normal kitting task. The “Robot_Normal” node checks conditions such as the operational status of the robot, the functionality of the gripper, and the risk of collision with other robots. Once these conditions are satisfied, the robot follows the sequence of actions planned by PDDL to complete the task. Specifically, the robot must move to the location of the target part, grab it, and then place it on the target AGV. If a failure occurs at any point, the behavior tree halts and is reconstructed based on the new task state to retry or recover from the error.

Figure 4b shows the behavior tree for a floor robot after recovering from a robot malfunction during an assembly task. Upon detecting the failure, the task is paused. After the failure in the behavior tree from Figure 4a, a new behavior tree, as shown in Figure 4b, is generated to retry and successfully complete the task.

Figure 4c demonstrates the behavior tree used by a floor robot when executing a part-flipping task. For a flipped part, the robot must first grab and flip it, then select a new surface to flip. The process is repeated until the flipping task is successfully completed.

Figure 4d shows the behavior tree for a ceiling robot performing a normal assembly task. The “Robot_Normal” node checks if the robot is functioning properly, including conditions for human safety. If the system detects proximity to a human, the robot switches to a “Far from Human” action sequence, ensuring safe operation before returning to the original task.

Figure 4e shows the behavior tree for a ceiling robot after recovering from a gripper malfunction during an assembly task. Upon detecting the failure, the task is paused. After the failure in the behavior tree from Figure 4d, a new behavior tree, as shown in Figure 4e, is generated to retry and successfully complete the task.

Figure 4f depicts the behavior tree for a ceiling robot executing a task that involves flipping a part. The robot must grab the part, flip it, and continue the task.

Real-time monitoring of conditional nodes via behavior trees ensures timely responses to agility challenges, overcoming issues such as reduced execution efficiency caused by delayed responses to agility challenges in traditional frameworks. A simple experimental demonstration of this framework can be found at: https://www.youtube.com/watch?v=QW96v9eKiBc, accessed on 21 August 2024.

### 4.2. Experimental Validation

To evaluate the performance of the proposed hierarchical robotic task framework in different scenarios, we conducted experiments on three types of tasks: kitting, assembly, and combined tasks. For each task type, nine sets of experiments were conducted, including one normal scenario and eight scenarios with various challenges. Each scenario was tested five times with random variations. The specific experimental setup is detailed in Table 3.

**Figure 5:**
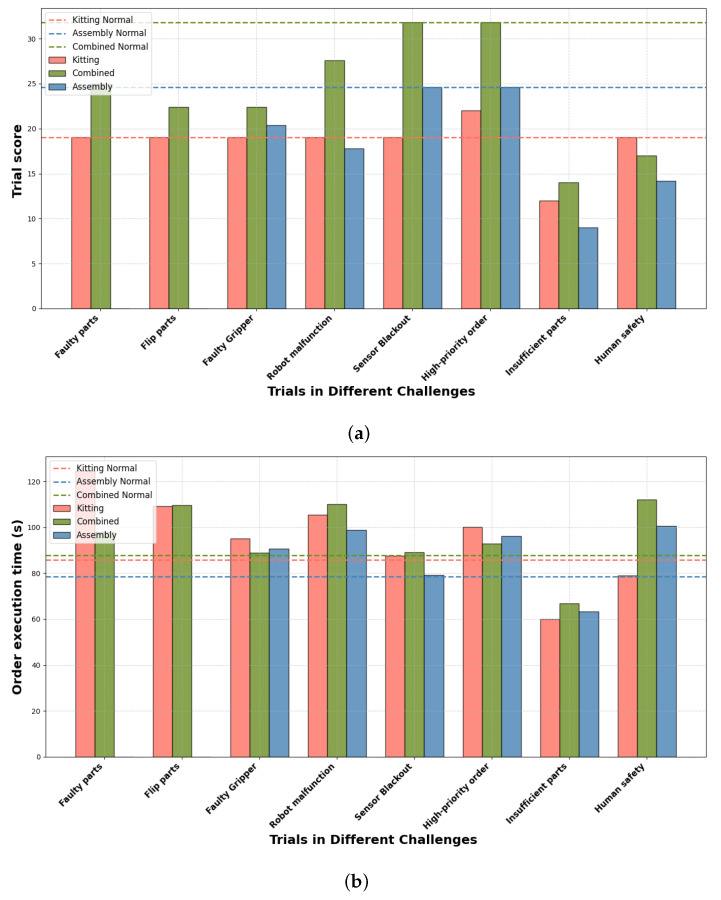
The results for various tasks and scenarios. (**a**) Trial score: the dashed line represents the baseline score in the normal state, whereas the bar chart represents the scores under various challenges. (**b**) Order execution time: the dashed line represents the baseline time in the normal state, whereas the bar chart represents the times under various challenges.

Figure 5 presents the experimental results across various tasks and scenarios, where task scores and task times serve as key performance indicators to evaluate the agility and effectiveness of the proposed hierarchical robotics task framework. Notably, the part poses in the assembly task are obtained through system calls; therefore, challenges related to faulty parts and flipped parts are not present. The high-priority order challenge divides the tasks into two orders, resulting in additional rewards that increase the scores beyond the baseline in a normal state.

The scoring formulas used in the experiments are as follows. The kitting task score $S_{k}$ is calculated as:

(1) $$S_{k}=\max\left({\mathrm{pt}}_{\mathrm{tray}}+\sum_{q}^{n}{\mathrm{pt}}_{q}+{\mathrm{pt}}_{b}-{\mathrm{pn}}_{ep},0\right)\times n_{des}$$

where ${\mathrm{pt}}_{\mathrm{tray}}$ represents the tray score, ${\mathrm{pt}}_{q}$ represents the score for each component, ${\mathrm{pt}}_{b}$ is the bonus score for successfully completing the entire task, ${\mathrm{pn}}_{ep}$ represents the penalty if more parts are on the tray than needed, and $n_{des}$ is the number of target locations.

The assembly and combined task scores $S_{a}$ and $S_{c}$ are calculated as:

(2) $$S_{a},S_{c}=\left(\sum_{s}^{n}{\mathrm{pt}}_{s}+{\mathrm{pt}}_{b}\right)\times n_{des}$$

where ${\mathrm{pt}}_{s}$ is the score for each component in the assembly or combined tasks, ${\mathrm{pt}}_{b}$ is the bonus score for successfully completing the entire task, and $n_{des}$ is the number of target locations.

In Figure 5a, the bar chart illustrates task performance across various challenges, with the dashed line representing the baseline score in a normal state. The results indicate that the framework excels at handling dynamic tasks, particularly in the kitting task, where the scores are consistently close to the baseline, even under challenging conditions. However, in the assembly and combined tasks, the scores show more variation, primarily due to the higher precision required for operations such as part insertion.

Figure 5b provides insight into task completion times. The increase in time compared to the baseline is largely due to the additional recovery steps necessary to handle faults or agility challenges, as seen in scenarios like robot malfunction or high-priority orders. However, the time increments remain relatively close to the baseline, demonstrating the system’s capacity for real-time fault monitoring and quick reactivity. The agent architecture, which integrates PDDL planning and behavior trees, plays a critical role in minimizing time overhead by dynamically adjusting to unexpected challenges and automatically updating the behavior tree during execution.

As discussed in Section 3.3.1, the planning time required by the PDDL approach was relatively minimal. Consequently, the primary factor contributing to the extended challenge time was the inclusion of additional recovery steps. Nevertheless, the overall completion time remained within acceptable limits. While this analysis predominantly addresses individual tasks, the proposed method exhibited consistent performance across multiple tasks. Notably, in the ARIAC 2023 finals, which required the simultaneous execution of various tasks, the scores accounted for sensor costs, task completion time, and task scores. Our approach ultimately secured first place in the competition. (Agile Robotics for Industrial Automation Competition|NIST, https://www.nist.gov/el/intelligent-systems-division-73500/agile-robotics-industrial-automation-competition, accessed on 28 August 2024).

## 5. Lessons and Conclusions

### 5.1. Lessons

From our experience of participating and winning awards for three consecutive years, the most important lesson we have learned is the choice of an agent-based approach. The structure of agents allows the system to be divided into independent units, each focusing on specific tasks. In ARIAC 2021 and ARIAC 2022, we used various functions as independent agents. In ARIAC 2023, we treated each robot as a complete agent. This modular design not only facilitated debugging and collaboration among team members but also significantly improved the system’s flexibility and scalability.

### 5.2. Conclusions

This paper details the hierarchical robot task framework adopted in ARIAC 2023. The framework employs an immediate task allocation strategy and integrates PDDL with behavior trees to form agents. The test results of ARIAC 2023 fully demonstrate the effectiveness of the system design. In addressing the challenges of flexible manufacturing tasks in dynamic and unpredictable environments, our approach focuses on rapid decision switching to adapt to dynamic challenges. Future work will concentrate on incorporating uncertainty into the planning process, integrating advanced techniques such as deep reinforcement learning to enable long-term decision-making in uncertain environments. For example, by integrating symbolic planning with reinforcement learning, as demonstrated in skill-based systems [31], our framework can leverage RL to enhance long-term decision-making capabilities by enabling agents to learn from dynamic challenges and optimize performance over time. Additionally, further exploration of the system’s scalability when dealing with larger teams of robots or more complex tasks would enhance the paper’s applicability to industrial-scale applications. By sharing our experience in ARIAC 2023, we hope to contribute to future ARIAC competitions and broader robotic applications.

## Figures and Tables

**Table 1:** Eight agile challenges in ARIAC 2023.

Challenge	Description
Faulty parts	Parts have quality problems and cannot be used in the competition.
Flip parts	Parts may be placed upside down and must be reoriented.
Faulty gripper	The gripper may drop parts at any time, even if it does not move.
Robot malfunction	The robot may fail under certain conditions in the test, causing it to stop moving, which must be addressed.
Sensor blackout	The sensor may stop sending data for a period.
High-priority order	The simulation must complete a high-priority order before the regular-priority order.
Insufficient parts	The workspace does not have enough parts to complete the order.
Human safety	To simulate human movement in the workspace, the robot must ensure that it maintains a safe distance from people; otherwise, it will be punished.

**Table 2:** Behavior tree node types.

Node Type	Symbol	Success	Failure	Running
Sequence	→	If all children succeed	If one child fails	If one child returns running
Fallback	?	If one child succeeds	If all children fail	If one child returns running
Parallel	⇉	If >M children succeed	If >N − M children fail	else
Action	shaded box	If completed	Fail to complete	During completion
Condition	white oval	If true	If false	Never

**Table 3:** The trial details.

Trial	Description
normal_kitting	1 regular order: performing kitting on AGV4 and shipping the AGV to the warehouse. Randomly selected 4 parts.
normal_assembly	1 regular order: performing assembly at as1. Randomly selected 4 parts.
normal_combined	1 regular order: performing assembly at as2. Randomly selected 4 parts.
Faulty parts	Randomly set one faulty part on the tray, only effective for kitting and combined tasks.
Flip parts	Randomly set two parts that need to be flipped, only effective for kitting and combined tasks.
Faulty Gripper	Randomly set a gripper malfunction that causes one part to be dropped.
Robot malfunction	Randomly set a robot malfunction for 20 s.
Sensor Blackout	Randomly set a sensor blackout for 20 s.
High-priority order	The same workload is generated by two orders: 1 regular order and 1 high-priority order.
Insufficient parts	Randomly omit a required part from the order.
Human safety	The human operator purposefully moves towards the ceiling robot to interfere with the robot’s current task.

## Data Availability

Data are contained within the article.
